# Organizing Technology Transfer by Foreign Medical Teams for Reduction of the Global Geographical Discrepancy of Cardiac Surgical Care

**DOI:** 10.21470/1678-9741-2023-0053

**Published:** 2023

**Authors:** Nazmul Hosain, Adanna Akujuo, Şeref Alp Küçüker

**Affiliations:** 1 Department of Cardiac Surgery, Chittagong Medical College, Chattogram, Bangladesh.; 2 Department of Cardiothoracic Surgery, Tower Health Medical Group, West Reading, Pennsylvania, United States of America.; 3 Department of Cardiovascular Surgery, Ankara Sehir Hospital, Turkey.

Technology transfer may be described as the process of disseminating technical knowledge from the origin to wider distribution among people and places. This is the process of moving intellectual capital of an institution. Examples of technology transfer include bridging the gap between the academia and the industry^[1]^. Technology transfer is best understood as a course of action by which “know-how” information called “technology” is transferred across a boundary or boundaries to another country or organization^[2]^. Efficient technology transfer would lead to efficient use of technology^[3]^. Cardiovascular surgery is one of the most difficult and sophisticated branches of medical science. There are innovative specialties of health arena that regularly recruit latest technological advancements in their armory of equipment and devices^[4]^. These will ensure dissemination of innovations to benefit maximum number of people all over the world. Technology transfer thus can be of great help in the field of medicine. The World Health Organization (WHO) states that transfer of health-related technology to developing countries can enable these recipients to produce products locally and may increase access to improved health^[5]^. This transfer can take several forms ranging from licensed agreement, provision of information, know-how, and performance skills to technical materials and equipment. One very effective mode of technology transfer involves visit of developing countries by medical teams from the developed World. This popular method is being used for capacity building of the physicians and surgeons of Third World countries by experts from the Western World. Technology transfer is a two-sided process; the effort is therefore made to uphold the concern of both sides. For technology transfer to be successful, both parties should achieve benefit and acknowledge each other’s interests. Concessions on both sides are inevitable to establish partnership and cooperation replacing conflict and misunderstanding^[6]^.

Organizing the visit of a foreign medical team for the purpose of education, training, or technology transfer is a challenging job. It requires hard work both on the part of the visitors and the recipient organizers, making it a highly specialized subject. Such visits, particularly when take place between two countries with diverse socioeconomic conditions, involve a number of difficult issues. Immigration, logistics, security, and patient selection all need to be taken care of. The notion of technology transfer between two industrialized countries is mostly straight forward, but it is much more fluid when transfer is made to a developing country^[3]^. There are several important issues of the recipient sides in Third World countries, which might not be known to the visiting teams. Without addressing these issues, the goals of technology transfer would not be achieved. Moreover, the process itself might turn out to be dangerous as well. Proper planning and preparation with the background knowledge of both the institutions can ensure the successful completion of technology transfer missions.

It is a natural tendency for every developing nation to try acquisition of latest technology for national development. But they often face social and cultural barriers in optimal utilization, adaptation, and dissemination of these skills. Technology transfer is a specialized and sophisticated process, and its structure often remains beyond the reach of the developing nations^[3]^. That’s why proper understanding of the status of the recipient country is crucial for the visiting team. It is also important for both the teams to understand in advance the difficulties involved in the procedure of technology transfer of their particular case.

There are some prime examples of technology transfer to Third World countries by the visiting surgeons. Perhaps one of the best examples is the introduction of cardiac surgery in India by Dr Reeve Hawkins Betts, an American missionary surgeon. He established the first Cardiothoracic Surgery Department of this region in Christian Medical College Vellore, at the Indian state of Tamil Nadu, back in 1948^[7]^. Dr Betts was a Second World War veteran surgeon with huge experience of managing battle field chest injuries while serving in Africa^[8]^. After the war, he remained in India for 12 long years defying many prestigious offers in his home country^[9]^. He trained the first generation of Indian cardiothoracic surgeons. This brilliant leader became the founder President of Indian Association of Cardiothoracic Surgeons, established in 1954. Dr Betts managed effective technology transfer to his Indian disciples, and till date he is well respected for his contribution in the development of cardiothoracic surgery in the region^[10]^. A similar role was attempted by a Canadian missionary surgeon, Dr Donald Edward Bowes, in neighboring Pakistan. Dr Bowes spent 15 years at the United Christian Hospital Lahore trying to establish cardiac surgery in Pakistan. He bought equipment, human resources, and appropriate technology in his effort^[11]^. But, somehow, he didn’t succeed in transferring the technology to the local surgeons and create a legacy. As a consequence, while Dr Betts is still regarded with respect as the founder of cardiac surgery in India, Dr Bowes is almost a forgotten. That shows the importance of importing technology and making proper transfer for capacity building of the competent local authority to create a long-lasting beneficial effect and legacy.

In real world, there is a wide discrepancy both in terms of quality and quantity of the cardiac surgical services provided in different countries. According to the information provided by the Society of Cardiothoracic Surgery of Great Britain and Ireland, the annual number of cardiac surgical procedures performed in the National Health Service hospitals ranged between 35000 & 40000 over the period of 2010-2013^[12]^. The total number of cardiac procedures in Japan was 60000 in 2011^[13]^. The population of Bangladesh is 2.5 and 1.25 times that of UK and Japan, respectively. But the total number of operations performed there was 9094 in 2015^[14]^ and 11121 in 2016^[15]^. In addition, there is gross discrepancy in quality of service as well. Far from the organized approach practiced in the West, cardiac surgery in the Third World is still being practiced on provisional basis. Insufficient resources, inexperienced manpower, and inadequate training programs are a few of the rate limiting factors to access the lion share of world’s population to most of these facilities. The organized visits of foreign medical teams for the purpose of technology transfer have been an important measure in disseminating updated knowledge and technology.

In this editorial, we share our long experience of organizing foreign medical team visits for the purpose of technology transfer and provide key points of those experiences to the possible opportunity visitors. Minute details of such visits will provide helpful insight for preparing upcoming overseas missions. Visiting team members from the developed countries might often have very little idea about the prevailing situations in the hospitals of the low- and middle-income countries, and this editorial can help them to prepare to face the possible unforeseen problems ahead of their trips. This can also help the recipient institutions to prepare for technology transfer.

## Authors' Background

With the capacity of the International Affairs Secretary of Bangladesh Medical Association and the Coordinator of Foreign Training at the National Institute of Cardiovascular Diseases (NICVD), the corresponding author was involved with hosting 14 foreign medical teams visiting Bangladesh for technology transfer between 1996 and 2013 (Table 1). These visits were hosted by NICVD and Chittagong Medical College & Hospital (Chittagong). The duration of these trips ranged from three days to one month. This has given the author a wide experience in managing such visits. Data from different visits were compiled from personal records, hospital official figures, government statistics, and other sources. Feedback from the visiting teams was also sought. Travel-related data were collected from travel agents, International Air Transport Association links, and various civil aviation sources.

**Table 1:** List of visitors to Bangladesh for technology transfer hosted by the corresponding author

Time	Venue	Country	Team leader/Key surgeon	Duration
1996	NICVD	France	Dr Akhtar Ali Rama, Hôpital Pitié-Salpêtrière, Paris	2 weeks
1996	NICVD	USA	Dr Alim Khondoker, Tennessee	2 weeks
1996	NICVD	USA	Dr Brad Vezales, Lancaster General Hospital, Pennsylvania	2 weeks
1997	NICVD	India	Dr M R Girinath, Apollo Hospital, Chennai	1 week
2003	NICVD	India	Dr Anil G Tendolkar, LTMC, Mumbai	1 month
July 2004	NICVD	India	Dr Arkalgud Samathkumar, AIIMS, Delhi	1 week
December 2004	NICVD	Malaysia	Dr Azhari Yakub, Institute Jantung Negara, Kuala Lumpur	1 week
December 2004	NICVD	USA	Dr Hormoz Azar, Eastern Virginia Medical School, Virginia	1 week
2005	NICVD	India	Dr Anil G Tendolkar, LTMC, Mumbai	1 month
December 2005	NICVD	USA	Dr M Abidur Rahman, Michigan State University	2 weeks
April 7-21, 2012	CMCH	India	Dr Anil G Tendolkar, LTMC, Mumbai	2 weeks
June 7-14, 2012	CMCH	India	Dr Anil G Tendolkar, LTMC, Mumbai	1 week
November 4-8, 2012	CMCH	Turkey	Dr Mustafa Paç, Specialized Hospital, Ankara	1 week
January 12-15, 2013	CMCH	USA	Dr Kim F Duncan, University of Nebraska Medical Center	3 days

AIIMS=All India Institute of Medical Sciences; CMCH=Chittagong Medical College & Hospital; LTMC=Lokmanya Tilak Municipal Medical College; NICVD=National Institute of Cardiovascular Diseases; USA=United States of America

The second author has been involved with organizing technology transfer missions operated to Africa. The third author has been part of technology transfer mission teams from Turkey to Azerbaijan and Uzbekistan. In this way, we analyze these issues from various perspectives.

## Preparations

The preparations related to technology transfer missions may be addressed under the following headings:

1. The need assessment of the recipient institution and sustainability
  Before beginning any mission, a proper need assessment of the recipient center must be carried out. Third World country hospitals are often disorganized and not aware of their actual deficiencies. If need assessment is not done properly on time, there may be setback in achieving the goals of technology transfer. A team of experts of the host center would best do the need assessment to identify the areas where technical cooperation is needed. It should be done in a manner so that different level of personnel may participate. Statisticians and Human Resource Departments are to be involved in this procedure for bringing the real picture as consultants often tend to hide their areas of weakness.
  Many low-income countries do not have a national healthcare system. Prior to initiation of the collaboration, a proper assessment must be made regarding means of sustainability of the program in between visits and after the final exit of the visiting team. A concerted effort should be made to seek a constant source of long-term support locally. Without this, a lot of effort might be placed into the attempted transfer of skills that will collapse once the resources provided by the visiting team no longer exist.
2. Suitable matching
  Finding a suitable team may not be easy as it seems. There has to be good matching of the recipient institution’s requirement and the visitor’s extent of offer. This matching involves different dimensions like the level of efficiency, timing, linguisticsocioeconomic issues, and willingness for cooperation. In our experience, finding a voluntary team willing to work on philanthropic basis gives the best chance of technology transfer. There are some voluntary organizations regularly involved in technology transfer. These regular performers are usually well prepared and effective. But getting a slot in their busy schedule may be a matter of difficulty. There are some other organizations, but those only perform the act of matchmaking between donor and recipient institutions. They usually engage third party experts for this purpose.
3. Immigration, government permission, and registration
  When transfer is between two different countries, the issues of immigration and government permission come. For movement of people across international border, passport and visa are standard practice. Since the visiting teams come from developed countries and go to developing countries, obtaining visa often appears an easy job. Sometimes visa is even not required or there is automatic waiver. But this is not the case always. Often someone would find difficulty in obtaining visa, particularly when non-government centers are involved. Also important is getting the correct type of visa for the trainers. Handling patients even for technology transfer with “tourist” or “visitor” category of visa might be a punishable offence in some countries. Sometimes it may take days, even months to process visa procedures. So, visa procedures should be started early.
  For proper technology transfer to take place, the visitors have to offer hands-on training. This would require permission from the Government of the recipient country. In addition, visiting medical personnel have to be registered by the host country authority responsible for registering doctors, perfusionists, and nurses. This very important aspect often tends to be ignored by the organizers. But this may become a very serious concern as litigation procedures are likely for violation of the local law. In some countries this may constitute a very serious criminal offense with severe punitive measure even for the visiting team as well as the organizers.
4. Coronavirus disease 2019 (COVID-19) concerns
  When the initial draft of this editorial was made, there was nothing as COVID-19. Since early 2020, the world has changed due to appearance of this disease, and this change is permanent. Numerous new regulations have been imposed by different countries on traveling, entry, and immigration. This has altered the medical tours for the purpose of technology transfer as well. In addition to passport, visa, and other standard travel documents, travelers now might also have to carry COVID-related papers, including vaccination certificates and recent COVID-19 test reports. These factors are to be taken into consideration while making travel plans. While entering a new country, the remote possibilities of isolation, quarantine, and hospitalization plans are also to be included in contingency planning. Moreover, COVID-19-related regulations may change suddenly. So, the last-minute change is also a possibility, it’s better to be prepared for that as well.
5. Arranging the logistic support
  A huge logistic support is needed for arranging such programs to ensure effective technology transfer. If the visitor and the host team members are ready, but hospital and patients are not, technology transfer might not take place. That is why all the logistics required for the professional service must be ensured and synchronized with the visit. The list of logistics includes the disposables, medicines, food, drinks, and other support materials. Without ensuring these logistics, the initiative may be a complete wastage. Both the recipient and donor team should coordinate ensuring logistics before any mission begins.
  The visitors bring a lot of materials with them, and these are often donated free of cost. But these free items might not turn free at the recipient countries. These may be subjected to tough customs regulations, inspection, and taxation. This is particularly true for unaccompanied luggage; customs officials usually have softer attitude towards baggage carried along with the team. If bulk amount of material is to be sent, using surface or sea transport may be cost effective, but it may require very long time for transportation. Some equipment or instruments which are brought by the visitors to be used during the mission and then taken back home on completion may be a matter of contention. The regulations related to import and re-export may be applicable. Early documentation in consultation with the host country authority is strongly advised to avoid big hassle later.
  While using or implanting biological material in patients, the local religious or cultural values should be kept in mind. Using porcine materials in a Muslim or bovine materials in a Hindu patient may be highly objectionable. So, the correct bio-derived items should be chosen keeping the destination in mind.
6. Patient selection and management
  Patient selection is an important issue. Proper selection of patients would ensure perfect technology transfer. Ideally, the patients should be those difficult to be managed by the host team but easily managed by the visitors. Such cases would ensure proper technology transfer. But it should be remembered that the diagnostic tools and methods available in the recipient country might not be acceptable for the visitors. One way of combating his problem is using the modern methods of communications like Zoom, Skype, WhatsApp, Viber, or the telemedicine apparatus in patient selection before the visiting team starts.
  On occasions it becomes a matter of opportunity and prestige for many patients in a Third World country when a visiting team from a developed country shows up. This may create serious chaos in patient selection often accentuated by political pressure.
7. Accommodation, meals, transport, and security
  The arrangement of meals and accommodation must match the requirements of the visiting team and the capability of the host. It is possible that the recipient team might not have any idea about the dietary pattern of the visitors. Special dietary requirement like vegetarian, halal, kosher, or glutenfree meals often have to be met. Timing of major meals is often a cultural issue with considerable variation among the countries concerned. Jet lag may affect the performance of team members from a country far of on a short trip.
  The tropical climate itself may be a big trouble for the visitors coming from the temperate zone. Although the operation theater and intensive care unit are supposed to be air-conditioned, the air conditioner may be out of order or inadequate in these recipient centers. The general wards are unlikely to be covered by air conditioning. Mosquitoes, flies, and other insects may be source of constant botheration. Traveler’s diarrhea is a very irritating condition for the visitors. The habit of carrying small dispenser of alcohol-based hand washing solutions may make it easier for the travelers wash their hands before eating^[14]^. The role of probiotics in the prevention of traveler’s diarrhea seems not that well established compared to other forms of diarrhea^[15]^. Certain species of bacteria can have large effects on the gut immune system, and the balance of these influences is important for maintenance of homeostasis^[16]^. Use of prebiotics and probiotics may be a powerful strategy for manipulating the microbial composition and immune response of the host. A useful practice adopted by many visitors is simply by having yogurt every morning. The nonpathogenic microorganism from the yogurt colonizes the gut and saves from the local flora, which may be pathogenic for the visitor. Drinking water is a big problem in Third World countries. Even some of the locally bottled mineral water might not be safe for drinking. Checking with the host which brand of mineral water is safe is always a good idea. Also, their advice helps in choosing local foods. Drinking repeated servings of light tea or green tea liquor is a safe way of taking plenty of safe fluid as their preparation involves boiling of water. Wherever possible, all vaccination should be ensured for the visitors adequately ahead of the visit schedule. Common vaccinations suggested before making short-term visits to the tropics include hepatitis A, typhoid, cholera, etc. A quick check of Centers for Disease Control and Prevention web page for traveler’s advice is a good idea. It is always handy to keep a box of common emergency medications ready for the team members themselves.
  There should be adequate arrangement of local transport. In today’s world, security is a big concern, and it must be addressed properly. A visiting medical team may be a soft target with huge publicity reward for a terrorist organization. Help should be sought from the local law enforcing agencies as well as the embassy of the visitors. It is mandatory to write the law enforcing authority early to give them time to ensure security.
8. Organizing education and technology transfer
  The major purpose of such a visit is to provide education and technology transfer to the local medical personnel. Lectures, seminars, workshops, and hands-on training are some important tools for this purpose. It is always better to check what kind of public address equipment is available at the host institute. Some institutes even might not have basic equipment, like a multimedia projector. The visitors can prepare better if they are aware of the host situation well ahead of the visit. As the indigenous technological capabilities of the developing countries are weak by default, they tend to import technology internationally^[17]^. Finally, feedback reports from the recipient institution members should be collected. The host organizers should present their observations, pros, and cons. The visitors often require proper documentation of their activities for regulatory purpose back home. Notifying the organizers about these requirements in advance is always a good idea.
  Proper interaction between the visiting and the hosts teams is very important for technology transfer. This was practiced by the Turkish team. Prior to their visits to Baku and Tashkent, they used to invite surgeons, anesthetists, and nurses from Azerbaijan and Uzbekistan and participate in their operations. These would create a rapport between the visiting and the host team members to facilitate easy technology transfer when the actual missions take place.
9. Budget and finance
  Budgeting is an important factor. The financial ability of the host in the Third World country should be taken into consideration, a budget must be prepared. The source of finance may be the government or non-governmental. The latter may include organizations like WHO, the United Nations Children’s Fund (or UNICEF), pharmaceutical industries, etc. Some visiting teams have enough source of fund, and they finance themselves. Whereas in other cases, the host country might have to arrange the resources. Corporate social responsibility funds, an obligatory spending for the big business houses, may sponsor such programs. There are a few international third-party donors, who may provide funds for these benevolent actions. Proper audit of the accounts is to be ensured because these cross-country missions often become matter of serious scrutiny by the government and the media.
10. Press and media
  In Third World countries, media coverage is another important aspect of such programs. The composition and schedule of the visiting team should reach the common people on time so that they can respond. This dissemination of information may be done through press conference, press release, advertisement, or fliers. This aspect should be planned in advance, so that the potential patients can enroll and take advantage of the program and hereby facilitate technology transfer. The success stories are also to be disseminated the same way for appreciation and future references. Media and press can play an important role in this regard.
11. Closing and preparation for the next program
  Finally, a nice closing program is to be organized. The organizers should produce a brief and precise report. And future plans are to be finalized. The visitors might require proper documentation and certification from the recipient authority. This is often a regulatory or financial obligation on their part. It should be remembered that technology transfer like all other learning is a continuous process, and whenever possible, these should be followed up by successive events until the ultimate purpose is served.
12. Continuation of training and exit plan
  The duration of visits usually ranges from one to four weeks. These often occur once or twice a year. There may be a period in between when the host team is not actively involved in surgical cases. Repetition and consistent exposure are the key to build competence. Consideration should be made for additional training prior to commencement of the collaborative effort. This will decrease the duration of time to independent surgical practice by the host team. Once deemed safe, the host team should begin to do very selected low-risk cases with virtual support by the visiting team. Higher-risk cases should be saved to be performed during the duration of time the visiting team is present.
  As efforts by the visiting team are rarely infinite, an exit plan should be formulated. This typically occurs when it is felt that the host team has acquired adequate competence. Support from the visiting team should be weaned preferably with continued virtual support as opposed to abrupt termination. Good outcomes will be vital for the survival of a successful program. An abrupt exit may lead to improper case selection by the host team and failure to rescue in the perioperative period. Adequate virtual support might obviate this.

Technology transfer in the medical field is the call of the day. The transfer of technology between the countries plays a key role in the global development. To reduce the disparity of accessibility to health care, technology transfer is an essential tool. Visit of a skilled team of experts from a developed country to a host in a developing country with less skilled medical manpower may produce very effective technology transfer. This ensures direct learning through hands-on training for capacity building in the field of medicine or surgery. The experts from the developed countries often volunteers for such missions in technology transfer in the least developed countries. The technological capabilities of the developing countries are a decisive factor in successful transfer and absorption of the technology. If carried out through proper planning and effective implementation, these missions become useful modes of technical and clinical development. But if not done properly, it might end up as a complete failure. It may become dangerous for the members of the mission as well. For this reason it is important for the visitors to know the pros and cons of management of such missions, particularly the issues from the host side, which are often unknown to them. Points figured out by an experienced host would help visitors prepare better for such future missions.
